# Immunoglobulins stimulate cultured Schwann cell maturation and promote their potential to induce axonal outgrowth

**DOI:** 10.1186/s12974-015-0331-7

**Published:** 2015-05-29

**Authors:** Nevena Tzekova, André Heinen, Sebastian Bunk, Corinna Hermann, Hans-Peter Hartung, Birgit Reipert, Patrick Küry

**Affiliations:** Department of Neurology, Medical Faculty, Heinrich-Heine-University, Moorenstrasse 5, 40225 Düsseldorf, Germany; Department of Immunology, Baxter Innovations GmbH, Vienna, Austria; Medical Affairs EMEA, Baxter Innovations GmbH, Vienna, Austria

**Keywords:** IVIG, Antibody, Differentiation, Interleukin-18, Myelin, Peripheral nerve, Regeneration

## Abstract

**Background:**

Schwann cells are the myelinating glial cells of the peripheral nervous system and exert important regenerative functions revealing them as central repair components of many peripheral nerve pathologies. Intravenous immunoglobulins (IVIG) are widely used to treat autoimmune and inflammatory diseases including immune-mediated neuropathies. Nevertheless, promotion of peripheral nerve regeneration is currently an unmet therapeutical goal. We therefore examined whether immunoglobulins affect glial cell homeostasis, differentiation, and Schwann cell dependent nerve regenerative processes.

**Methods:**

The responses of different primary Schwann cell culture models to IVIG were investigated: immature or differentiation competent Schwann cells, myelinating neuron/glial cocultures, and dorsal root ganglion explants. Immature or differentiating Schwann cells were used to study cellular proliferation, morphology, and gene/protein expression. Myelination rates were determined using myelinating neuron/glia cocultures, whereas axonal outgrowth was assessed using non-myelinating dorsal root ganglion explants.

**Results:**

We found that IVIG specifically bind to Schwann cells and detected CD64 Fc receptor expression on their surface. In response to IVIG binding, Schwann cells reduced proliferation rates and accelerated growth of cellular protrusions. Furthermore, we observed that IVIG treatment transiently boosts myelin gene expression and myelination-related signaling pathways of immature cells, whereas in differentiating Schwann cells, myelin expression is enhanced on a long-term scale. Importantly, myelin gene upregulation was not detected upon application of IgG1 control antibodies. In addition, we demonstrate for the first time that Schwann cells secrete interleukin-18 upon IVIG stimulation and that this cytokine instructs these cells to promote axonal growth.

**Conclusions:**

We conclude that IVIG can positively influence the Schwann cell differentiation process and that it enhances their regenerative potential.

## Background

Schwann cells, the myelinating glial cells of the peripheral nervous system (PNS), play a crucial role in the generation, function and maintenance of peripheral nerves. They provide electric insulation by the formation of lipid-rich myelin sheaths around large caliber axons. Schwann cells are also key players in nerve regeneration processes as they can quickly adapt to pathological situations, dedifferentiate, undergo proliferation, promote axonal regeneration, redifferentiate, and restore myelin sheaths [1]. In the injured PNS, a repair response is initiated by Schwann cells in which they degrade and phagocytose extracellular myelin debris, attract macrophages for further clearance, and provide axons a supportive and attractive environment to grow along [2, 3]. A large number of studies also demonstrated that Schwann cells can interact with cells of the immune system and that they exhibit immune cell properties, comprising antigen presentation, secretion of cyto- and chemokines, expression of complement components, and surface receptors involved in pathogen recognition (reviewed in [4]). This immune competence along with the plastic differentiation potential of Schwann cells provide the PNS with a certain regeneration capacity and further indicates that these glial cells are central components of many, if not all, nerve pathologies. Nevertheless, nerve regeneration in the PNS faces limitations, in particular when it comes to inflammatory and inherited neuropathies, or in peripheral nerve pathologies induced by toxins, drugs, or in diabetic patients [5–8]. For most of these neurological conditions, functional restoration of damaged axonal tracts and myelin sheaths represents the ultimate therapeutic goal which is, however, currently unmet. Intravenous immunoglobulins (IVIG) are generally used for the treatment of immune deficiencies. Moreover, IVIG are considered as first-choice therapy for acute or chronic polyneuropathies [9]. IVIG consist of polyclonal human immunoglobulin G (IgG) which is purified from large pools of human plasma donations. IVIG’s exact mode of action in the treatment of demyelinating autoimmune diseases still needs to be defined but is most likely based on several immunomodulatory mechanisms such as interaction with Fc receptors (fragment crystallisable (Fc)), binding to pathogenic antibodies/anti-idiotype effect, inhibition of complement binding, suppression of proinflammatory cytokines as well as modulation of T and B cells [9]. Therapeutic effects of IVIG have also been demonstrated in demyelinating disease models of the PNS, e.g., experimental autoimmune neuritis (EAN). Treatment with IVIG resulted in the reduction of disease severity and accelerated recovery [10, 11]. Although no current evidence exists indicating that IVIG also directly affect Schwann cells, recent data point to a role of endogenous antibodies in rapid myelin clearance and axon regeneration after nerve injury [12].

In the central nervous system (CNS), however, immunoglobulins were shown to influence oligodendroglial cells in that monoclonal oligodendrocyte-reactive immunoglobulin M (IgM) antibodies can enhance remyelination [13, 14]. Moreover, treatment of demyelinating disease models with polyclonal human IgG and IgM molecules directly enhanced oligodendrocyte differentiation [15–17]. Fc receptors for IgM were found to be expressed on mouse oligodendrocytes, their precursor cells, and myelin, which suggests that IgM directly interacts with glial cells [18].

Based on these observations, the aim of this study was to determine whether immunoglobulins, in particular IVIG, can directly interact with Schwann cells, whether this influences cell homeostasis and maturation, and could thus be used to promote nerve regeneration in different nerve pathologies. Using different models of cultured non-differentiating and differentiating Schwann cells, we found that these cells are not only able to bind IVIG but also respond to this stimulation with a promoted maturation reaction as well as an increased potential to support axonal outgrowth.

## Methods

### Schwann cell culture

Primary rat Schwann cells were isolated from Wistar rat neonatal sciatic nerves according to previously published protocols [19]. Schwann cells were grown on poly-D-lysine (PDL; Sigma-Aldrich, Taufkirchen, Germany) coated flasks, dishes or glass coverslips in Dulbecco’s modified Eagle’s medium (DMEM) low glucose (Gibco, Karlsruhe, Germany) supplemented with 10 % fetal bovine serum (FBS; Lonza, Cologne, Germany), 2 mM L-glutamine (Gibco), penicillin/streptomycine (Gibco), and 2 μM forskolin (Sigma-Aldrich). shRNA-mediated suppression of the p57kip2 gene was done using a nucleofector II device (Lonza, Basel, Switzerland), positively selected by means of hygromycin resistance plasmid, all as previously described [20]. Cells were kept for up to 9 days in medium supplemented with 50 μg/ml hygromycin B (Invitrogen, Karlsruhe, Germany). Cellular proliferation rates were determined either via 5-bromo-2′-deoxy-uridine (BrdU; Roche Applied Sciences, Penzberg, Germany) incorporation or by their Ki67 immunopositivity. For the analysis of Schwann cell growth, IVIG and control buffer were added to the culture medium 2 days post transfection and treatment was pursued for 3 days under selection conditions. Morphological measurement of cell processes was done on living cells (expressing fluorescent citrine protein, green) on a Nikon Eclipse TE 200 microscope using NIS-Elements AR 3.10 software (Nikon Instruments Europe BV, Amsterdam, Netherlands).

### Myelinating neuron/glia coculture

Mouse dorsal root ganglion (DRG) cultures were prepared from embryonic day 15 (E15) C57BL6 mouse embryos according to a previously published protocol [21]. DRG dissociation was performed in 0.05 % trypsin (Gibco) and 0.05 % collagenase (Sigma-Aldrich) for 20 min at 37 °C. Dissociated DRGs were resuspended in 40 μg/ml deoxyribonuclease I (Sigma-Aldrich), and the digestion was stopped with L15 Leibovitz’s medium (Gibco) containing 10 % horse serum (Gibco). For each condition approximately 500000 cells were seeded onto one collagen (Becton-Dickinson, Franklin Lakes, NJU, USA) coated x-well tissue culture chamber (Sarstedt, Nümbrecht, Germany) and incubated in growth medium at 10 % CO_2_ for 4 to 5 days. Myelination was induced by changing to mouse myelination medium [21] supplemented with 20 mg/ml dialysed IVIG or dialysed control buffer. After 7 days of buffer/IVIG treatment, cultures were fixed with 4 % paraformaldehyde (PFA, Merck, Darmstadt, Germany) and stained against myelin basic protein (MBP) and β-tubulin (TUJ1). MBP positive internodes were counted in both conditions and the average number was determined.

### Non-myelinating DRG explant cultures

DRG were dissected from E17/E18 Wistar rat embryos, cut into two to four explants and plated on glass coverslips coated with 1 mg/ml PDL and 13 μg/ml laminin (Sigma-Aldrich). Explants were cultured for 24 h as follows: (a) non-conditioned Schwann cell medium alone or supplemented with different concentrations of rat recombinant interleukin-18 (rrIL-18 10 ng/ml, 25 ng/ml, and 50 ng/ml; R&D Systems, Wiesbaden-Nordenstadt, Germany); (b) Schwann cell conditioned medium alone or Schwann cells treated with 10 ng/ml, 25 ng/ml, or 50 ng/ml rrIL-18 for 3 days; and (c) conditioned medium of Schwann cells treated with either dialysed control buffer or 20 mg/ml dialysed IVIG preparations for 3 days. Axons were visualized using neurofilament (NF) immunofluorescence. For quantification of axon outgrowth, microimages were taken (AxioVision software; C. Zeiss, Mainz, Germany) and axonal length was measured from the borders of the inner DRG core to the maximal axon spread using ImageJ software (National Institutes of Health, USA).

### Animal research according to ARRIVE guidelines

For this study, no animal in vivo experiments were performed. For the generation of primary cell cultures derived from embryonic and neonatal rats and mice (animal sacrifice and tissue elevation), we received approval by the local animal facility of the Heinrich-Heine-University (registration numbers O82/12, O69/2011, O53/11) in compliance with the German Animal Protection law (State Office, Environmental and Consumer Protection of North Rhine-Westphalia, LANUV NRW). For primary Schwann cells, we used P0 (postnatal day 0) Wistar rats of either sex; for myelinating neuron/glia cocultures, we used E15 (embryonic day 15) C57BL6 mouse embryos of either sex; and for non-myelinating DRG explant cultures, E17/18 (embryonic day 17/18) Wistar rat embryos of either sex were used. Adult C57BL6 mice and Wistar rats giving rise to pups and embryos were grown and kept in the local animal facility. Animals were housed under standard conditions with a 12 h light/12 h dark cycle. Water and food were available ad libitum.

### Immunoglobulin preparations

Gammagard Liquid (Immune Globulin Intravenous (Human)) 10 % containing 100 mg/ml protein was used (Baxter Healthcare Corporation, Westlake Village, CA, USA). The majority of the experiments were performed with IVIG lot LE12J270AB. Of note, two further IVIG lots (LE12L068AB; LE12L341AA) were tested and showed similar results in binding assays and functional analyses. In all experiments, IVIG were used at a final concentration of 20 mg/ml protein. Gammagard Liquid formulation buffer (0.25 M glycine, pH 4.5) was included as a corresponding control. F(ab′)_2_ fragments (fragment antigen-binding (Fab)) were generated from Gammagard Liquid (lot LE12L341AA) by incubation with pepsin (Sigma-Aldrich) at pH 4 and 37 °C under pyrogenic-free conditions. The reaction was halted by changing to pH 7, then the F(ab′)_2_ fragments were purified using size exclusion chromatography, filtered and adjusted to a final concentration of 31 mg/ml in 0.25 M glycine buffer, pH 4.5. The resulting F(ab′)_2_ fraction revealed an endotoxin concentration of 0.436 EU/ml and 2.3 % of Fc binding in comparison to IVIG was determined. The following human monoclonal antibodies were used as IgG1 controls: Avastin (Roche Pharma, Grenzach-Wyhlen, Germany), Herceptin (Roche Pharma), and Synagis (Abbott SRL, Campoverde di Aprilia, Italy) all at final concentrations of 20 mg/ml.

Prior to application, all antibody preparations (IVIG, F(ab′)_2_, IgG1), as well as the corresponding control buffers, were dialysed overnight at 4 °C to non-supplemented DMEM using Slide-A-Lyzer Dialysis Cassette 3.5K MWCO (molecular weight cut-off; Thermo Scientific, Rockford, IL, USA) according to the manufacturer’s protocol. Dialysis was performed in order to balance minor effects, which resulted from the IVIG formulation buffer. Of note, the distribution of IgG monomers, dimers, and oligomers was analyzed in IVIG preparations before and after dialysis by size exclusion chromatography (SEC-HPLC). An average of 89.35 ± 1.16 % monomers, 10.53 ± 1.14 % dimers, and 0.12 ± 0.02 % oligomers was found, which changed to 87.32 ± 1.97 % monomers, 12.78 ± 1.66 % dimers, and 0.15 ±0.05% oligomers after dialysis and subsequent storage of IVIG in cell culture media for 1–7 weeks. However, a number of initial experiments were performed with non-dialysed IVIG and revealed them to be as effective as dialysed ones regarding proliferation, gene expression, and morphological maturation. For IVIG binding studies, Schwann cells were plated onto glass coverslips, decorated with IVIG and control buffer for 24 hours in culture, then fixed with 4 % PFA and subjected to immunostaining. Decoration of Schwann cells with purified F(ab′)_2_ fragments was performed for 4 h. Purified F(ab′)_2_ fragments were used at an equimolar concentration corresponding to 5 mg/ml IVIG. The low molar concentration was used to prevent aggregate formation. Immunocytochemical staining and visualization were performed directly after cell fixation applying Cy3- or Alexa Fluor 488-conjugated secondary antibodies specific to human F(ab′)_2_ or human Fc gamma fragments (all Jackson Immuno Research Laboratories, Suffolk, UK) for 4 h at room temperature. Nuclei were visualized by means of 4′,6-diamidino-2-phenylindole (DAPI).

### RNA isolation, cDNA synthesis, and quantitative RT-PCR analysis

Total RNA was extracted from rat cultured Schwann cells using RNeasy Mini Kit (Qiagen, Hilden, Germany), and cDNA synthesis was performed by means of the High Capacity cDNA reverse transcription kit (Applied Biosystems, Darmstadt, Germany) using random hexamer primers. Quantitative RT-PCR gene expression measurement was assessed using an ABI PRISM 7900HT sequence detection system and SybrGreen universal master mix (Applied Biosystems). Relative gene expression levels were determined according to the ΔΔCt method (Applied Biosystems) using both glyceraldehyde-3-phosphate dehydrogenase (GAPDH) and ornithine decarboxylase (ODC) as reference genes. Specific primer sequences were designed with the Primer Express 3.0.1 software (Applied Biosystems) and tested for specificity by amplicon melt curve analysis. Primer sequences for the detection of GAPDH, ODC, MBP, Pmp22 (peripheral myelin protein-22), Sox10 (SRY-related HMGbox-10), Krox20/Egr2 (early growth response-2), and Oct6 (octamer-binding transcription factor-6) have previously been published [20] whereas the following additional primers were used: myelin protein zero (Mpz/P0) P0_fwd ACCTTCAAGGAGCGCATCC; P0_rev GCCATCCTTCCAGCTAGGGT; periaxin (Prx) Prx_fwd CGCCCGTGTGTTCTTTGAG; Prx_rev AGGGCTCGGCACATTGC; peripheral myelin protein 2 (Pmp2) Pmp2_fwd TGCAGAAGTGGGATGGTAAAGA; Pmp2_rev TCCACTACCATTTTCCCATCCA; proteolipid protein 1 (Plp1) Plp1_fwd CTTTGGAGCGGGTGTGTCAT; Plp1_rev TGTCGGGATGTCCTAGCCAT; connexin 32 (Cx32) Cx32_fwd CCTCCGGCATCTGCATTATC; Cx32_rev AGGCCCGGATGATGAGGTA; myelin associated glycoprotein (Mag) Mag_fwd CGCCTTTGCCATCCTGATT; Mag_rev TGTGACGTTCTTTTTTCTTCTTGTCT; monocyte attractant protein-1 (Mcp-1) Mcp-1_fwd ATGATCCCAATGAGTCGGCT; Mcp-1_rev CCTGCTGCTGGTGATTCTCTT; interleukin-18 (IL-18) IL-18_fwd TGTGTTCGAGGACATGCCTG; IL-18_rev GTCTGGGATTCGTTGGCTGT; Fc fragment of IgG, high affinity 1a, receptor (Fcgr1a/CD64) Fcgr1a_fwd TGGATCATACTGGTGCGAGGTA; Fcgr1a_rev TTGGTGCTGCGCTTAAGGA.

### Immunostaining

Schwann cells, myelinating neuron/glia cocultures, and DRG explants were fixed with 4 % PFA. Unspecific staining was blocked with either 2 % normal rabbit serum (NRS; Invitrogen) or 10 % normal goat serum (NGS; Sigma-Aldrich) in 0.5 % bovine serum albumin (BSA, Carl Roth, Karlsruhe, Germany) and 0.5 % Triton X100 (Sigma-Aldrich). Cells were then incubated with primary antibodies in phosphate-buffered saline (PBS; Sigma-Aldrich) supplemented with 2 % NRS/NGS overnight at 4 °C. Myelinating neuron/glia cocultures were blocked in 10 % NGS with 1 % Triton X100 and incubated with primary antibodies in PBS supplemented with 10 % NGS and 0.1 % Triton X100 overnight at 4 °C. DRG explants were blocked in 5 % low background solution (Inotech Biosystems, Brandon, FL, USA) and 0.1 % Triton X100 and then incubated with primary antibodies diluted in the same blocking buffer overnight at 4 °C. The following primary antibodies were used: goat anti-CD64 (R&D Systems; 1:500), rabbit anti-Ki67 (Abcam, Cambridge, UK; 1:100), rabbit anti-cleaved caspase 3 (Cell Signaling Technology, Boston, MA, USA; 1:100), mouse anti-MBP (Covance, Emeryville, CA, USA; 1:500), rabbit anti-β-tubulin (Covance; 1:1000), and mouse anti-neurofilament (Covance; 1:1000). Alexa Fluor 488- or Alexa Fluor 594-conjugated secondary antibodies (Invitrogen) were used for visualization of the signal. They were applied for 2 h at a dilution of 1:500 in PBS. Nuclei were visualized using DAPI. Stained cell cultures were analyzed using an Axioplan 2 fluorescence microscope (Zeiss, Jena, Germany) and Axiovision 4.2 software (Zeiss).

### Western blot analysis

Harvested cell pellets were lysed on ice with RIPA buffer (Cell Signaling Technology) supplemented with Halt protease and phosphatase inhibitor cocktail (Thermo Scientific). Whole cell lysates were subjected to two sonification cycles of 15 s each and then centrifuged at 14000×*g* for 10 min to obtain the soluble protein fraction. Protein concentrations were determined using the DC Protein Assay (BioRad, Munich, Germany). Samples were subjected to standard sodium dodecyl sulfate (SDS) gel electrophoresis and Western blotting using 4–12 % RunBlue SDS gels (Expedeon, Cambridgeshire, UK) and RunBlue Blot Sandwich nitrocellulose (Expedeon) following application of goat anti-CD64 (R&D Systems; 1:1000), mouse anti-GAPDH (Millipore, Temecula, CA, USA; 1:1000 for Odyssey detection/1:4000 for horse-radish-peroxidase (HRP) detection), mouse anti-P0 ([22]; 1:500), rabbit anti-MBP (Millipore; 1:500), rabbit anti-p38 mitogen-activated protein kinase (p38MAPK) (1:1000), rabbit anti-phospho-p38MAPK (1:400), rabbit ant-phosphatase and tensin homolog (PTEN) (1:2000), rabbit anti-phospho-PTEN (1:1000), rabbit anti-serine-threonine kinase (Akt) (1:4000), rabbit anti-phospho-Akt (1:500), rabbit anti-c-Jun (1:1000), rabbit anti-phospho-c-Jun (1:500) (all from Cell Signaling Technology), and mouse anti-actin (Becton-Dickinson; 1:1000) primary antibodies. Visualization of signals using IRDye 800CW donkey anti-mouse (1:15000) and IRDye 800CW goat anti-rabbit antibodies (1:10000) was done using an Odyssey infrared imaging system scanner (both LI-COR Biosciences, Lincoln, NE, USA). For visualization of signals using peroxidase-labeled horse anti-mouse (Vector Laboratories, Burlingame, CA, USA; 1:5000), peroxidase-labeled horse anti-goat (Vector Laboratories; 1:2000), or HRP-linked goat anti-rabbit (Cell Signaling Technology; 1:2000) secondary antibodies, nitrocellulose membranes were incubated for 5 min with SuperSignal West Pico Chemiluminescent Substrate (Thermo Scientific) and then exposed to a Amersham Hyperfilm ECL (GE Healthcare, Buckinghamshire, UK) to detect the HRP signal. Protein band quantifications were performed using the Odyssey software. The intensity for each band was determined and normalized to the intensity of the GAPDH band of the corresponding probe.

### ELISA

Culture media were collected and centrifuged at 2000 rpm for 10 min (4 °C), frozen on dry ice, and stored at −80 °C prior further analysis. ELISA measurements were performed with non-diluted media supernatants using the rat interleukin-18 ELISA kit (Invitrogen). Detection of the optical density was done at 450 nm on an Infinite M200 Pro plate reader (TECAN, Crailsheim, Germany).

### Statistical analysis

Data are presented as mean +/− standard error of the mean (SEM), and significance was assessed by two-sided Student’s *t* test, unpaired comparison for means (GraphPad Prism). Experimental groups were considered significantly different at *p<0.05, **p<0.01, ***p<0.001; ns, not significant. *n* represents the number of independent experiments.

## Results

### Human immunoglobulins specifically bind to the rat Schwann cell surface

**Fig. 1:**
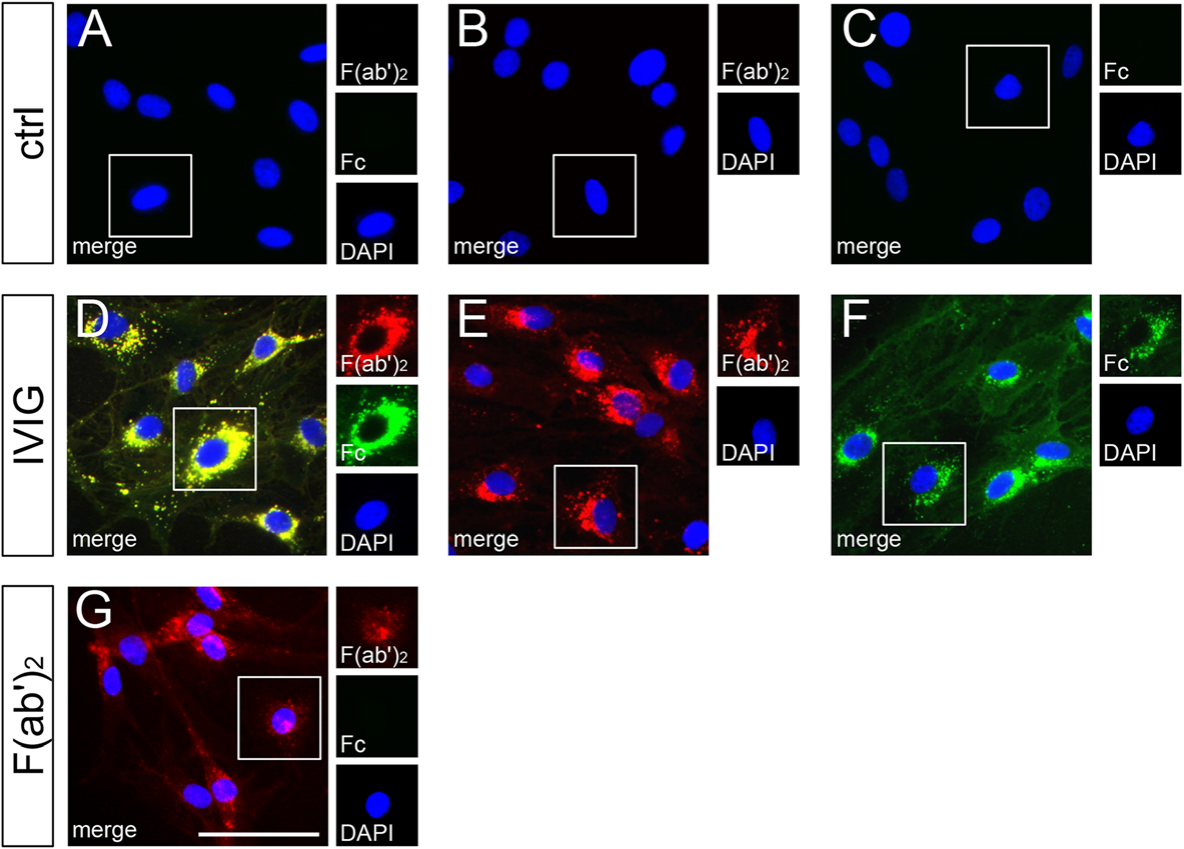
IVIG bind specifically to the Schwann cell surface. **a**–**g** Living Schwann cells were decorated for 24 h with either dialysed control buffer (*ctrl*) (**a**–**c**), 20 mg/ml dialysed IVIG (**d**–**f**), or dialysed F(ab′)_2_ fragments, corresponding to 5 mg/ml IVIG (**g**). After fixation, the cells were stained against human F(ab′)_2_ (*red*) and human Fc parts (*green*), and nuclei were labeled with DAPI (*blue*). IVIG and F(ab′)_2_ fragments binding could be detected by means of co-immunostaining (**a**, **d**, **g**) or single staining against human F(ab′)_2_ (**b**, **e**) or human Fc fragments (**c**, **f**). Application of dialysed control buffer (**a**–**c**) resulted in no staining. Scale bar, 50 μm. *n* = 8 for **a**–**f**, *n* = 4 for **g**

Several studies indicate that immunoglobulins may directly affect oligodendroglial cells, the myelinating glial cells of the CNS. This interaction was shown to promote cellular differentiation as well as remyelination [14, 16, 17] likely based on the expression of an IgM-specific Fc receptor on oligodendrocytes, their precursor cells, and myelin [18]. These observations prompted us to address the question whether immunoglobulins also bind to Schwann cell surfaces. To this end, we used a dialysed human IVIG preparation for the decoration of alive, non-differentiating primary rat Schwann cells in culture. By applying anti-human F(ab′)_2_-specific and anti-human Fc gamma-specific antibodies (i.e., F(ab′)_2_ fragments), we could demonstrate that human immunoglobulins specifically bind on the Schwann cell surface (Fig. 1a–f). Immunofluorescent signals corresponding to surface bound immunoglobulins could mainly be detected around perinuclear regions. Similar binding patterns were observed upon decoration of differentiating Schwann cells (data not shown), induced by means of suppression of the p57kip2 gene [20], as well as when further IVIG lots were used (data not shown). In order to study the binding mode we used a preparation of pepsin digested IVIG, which is enriched in human F(ab′)_2_ fragments, for Schwann cell decoration. These assays revealed a related binding pattern (Fig. 1g) indicating that it could be mediated via the Fab domains of the immunoglobulins. As further controls, we then used two human monoclonal IgG1 antibodies (Avastin and Herceptin) for decoration, revealing similar binding patterns on the Schwann cell surface (data not shown). Altogether, these observations suggest that IVIG binding (representing a large number of different IgG of different specificities) could take place either via Fab parts recognizing Schwann cell epitopes or via Fc parts interacting with Fc gamma receptors.

### Schwann cells express CD64 encoding a high affinity immunoglobulin receptor

**Fig. 2:**
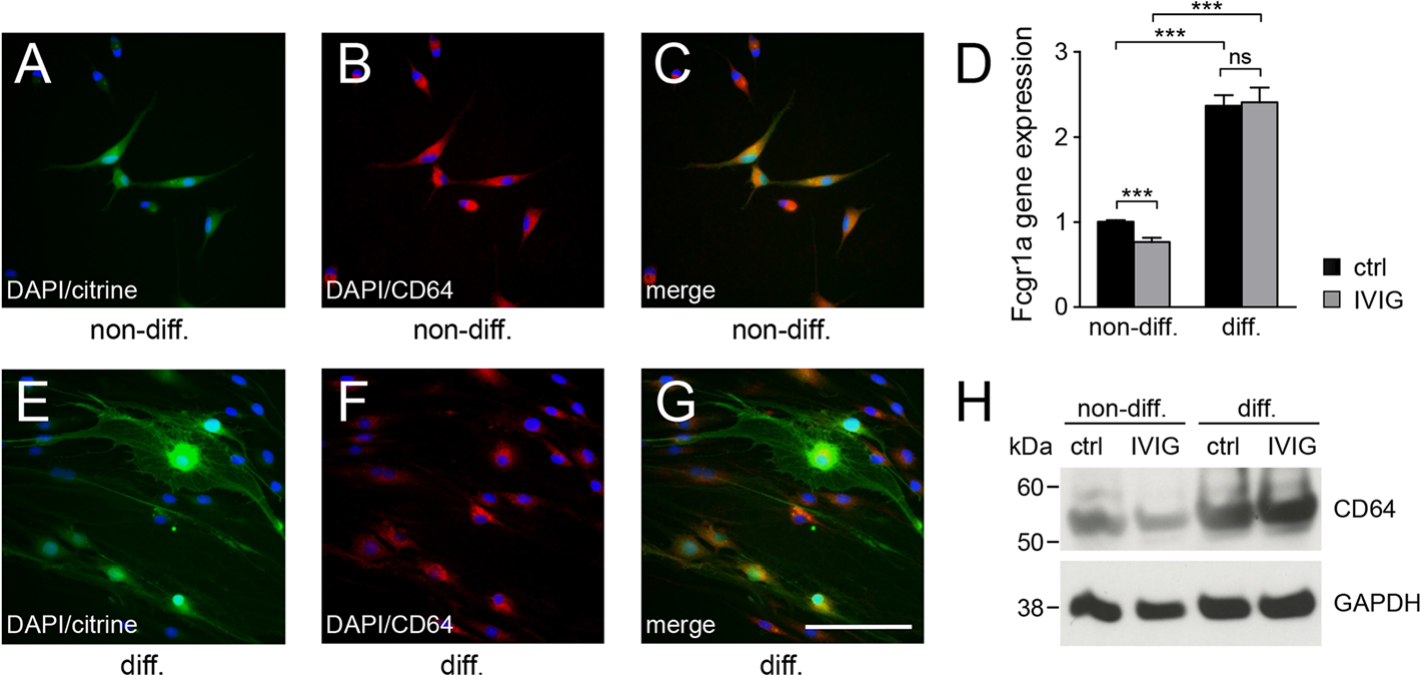
Schwann cells express the Fc gamma receptor CD64. On the surface of non-differentiating (*non-diff*.; **a**–**c**) as well as differentiating Schwann cells (*diff*.; **e**–**g**), CD64 protein expression was detected. Differentiation was induced by means of p57kip2 suppression, and modulated cells were visualized by means of citrine (*green-fluorescent* protein) expression. Cell nuclei were visualized by DAPI (*blue*). **d** Significant induction of rat Fcgr1a gene expression (*CD64*) under differentiation promoting conditions as determined by quantitative RT-PCR. Seven-day stimulation with 20 mg/ml dialysed IVIG (*gray bars*) in comparison to control buffer (*ctrl*; *black bars*) decreased significantly the expression of CD64 in non-differentiating Schwann cells, only (**d**). **h** Western blot analysis of differentiating and non-differentiating Schwann cells, stimulated with both IVIG and control buffer (*ctrl*) confirmed CD64 protein expression. Protein molecular weights are indicated in *kDa* (kilodalton). GAPDH expression was used for normalization. *t* test (*ns*, not significant, ***p < 0.001). *Error bars* represent SEM. Scale bar, 100 μm. *n* = 7 for **a**–**c**, *n* = 6 for **e**–**g**, *n* = 8 for **d**, *n* = 4 for **h**

Previous studies indicated that Fc gamma receptors (Fcgr), in particular Fcgr3/CD16, can be found on human and rat Schwann cells [23–25]. To further characterize Fc receptor expression by rat Schwann cells, we performed quantitative RT-PCR measurements of Fcgr1a/CD64, Fcgr2a/CD32a, Fcgr2b/CD32b, and Fcgr3a/CD16 transcripts. Interestingly, we could only detect expression of CD64 and furthermore found it to be significantly upregulated in differentiation competent rat Schwann cells (Fig. 2d). Notably, IVIG stimulation led to a small downregulation of CD64 expression levels in non-differentiating Schwann cells, whereas in differentiating cells CD64 expression was not influenced. We next performed staining experiments on non-permeabilized Schwann cells using a polyclonal CD64 antibody in order to confirm CD64 protein expression on the cell surface. Specific staining signals were distributed all over the surface of non-differentiating cells (Fig. 2a–c) whereas staining on differentiating Schwann cells was mainly located on the cell soma above the perinuclear region (Fig. 2e–g). The same polyclonal CD64 antibody was then applied in Western blot analysis confirming CD64 expression and upregulation in differentiating cells (Fig. 2h). Of note, the antibody was found to be specific as it also recognized a recombinant truncated version of rat CD64 (data not shown) as well as a band of the same apparent molecular weight when full-length Fcgr1a/CD64 with fused flag-tag was overexpressed in cultured Schwann cells (data not shown).

### IVIG stimulation affects Schwann cell proliferation and growth of cellular processes

**Fig. 3:**
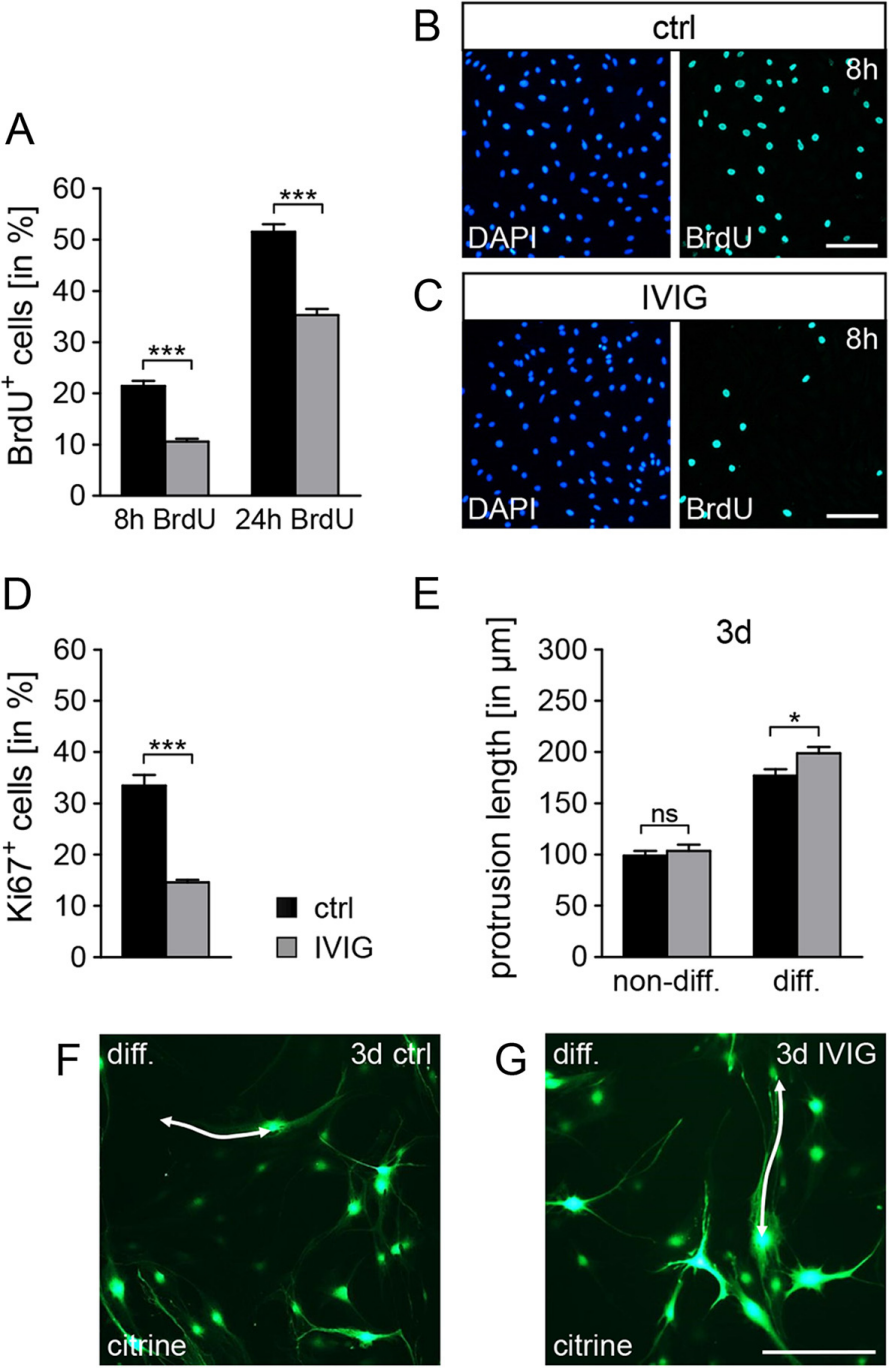
IVIG reduce proliferation rates and accelerate growth of cellular protrusions in differentiation competent Schwann cells. **a**–**d** Schwann cell proliferation is reduced after 2 days of stimulation with 20 mg/ml dialysed IVIG (*gray bars*) compared to control buffer (*ctrl*) preparations (*black bars*). **a** BrdU labeling was performed in the last 8 or 24 h (*8h*, *24h*) of the IVIG treatment and evaluation of the percentage of BrdU/DAPI double positive (*BrdU+*) nuclei compared to total number of DAPI nuclei revealed a significant reduction of proliferating cells. **b**, **c** Representative photographs of DAPI (*blue*) and BrdU (*green*) labeled nuclei after 8 h BrdU pulse of control buffer **b** and IVIG **c** stimulated cells. **d** Staining against the proliferation markers Ki67 and determination of the percentage of Ki67/DAPI double positive (*Ki67+*) nuclei in relation to the total number of DAPI positive nuclei. **e**–**g** Quantification of the average Schwann cell protrusion lengths revealed significantly accelerated growth of cellular processes in early stages of the differentiation process. **e** The cell processes of p57kip2 suppressed cells (*diff.*) were found to be significantly longer after 3 days (*3d*) IVIG stimulation when compared to control buffer treated cells. However, no difference was observed in non-differentiating (*non-diff.*) cells. **f**, **g** Representative photographs of differentiating citrine positive Schwann cells after 3 days control buffer (**f**) or IVIG (**g**) treatment; cell processes are marked by *white arrows. t* test (*ns*, not significant, *p < 0.05, ***p < 0.001). *Error bars* represent SEM. Scale bars, 100 μm in **b**, **c**; 200 μm in **g**. *n* = 4 for **a**–**c**, *n* = 2 for **d**, *n* = 10 for **e**–**g**

As a next step, we investigated whether IVIG exert an effect on primary Schwann cell proliferation. Stimulation with dialysed IVIG revealed a significant reduction of the proliferation rate of non-differentiating cells as revealed by the reduction of incorporated BrdU (Fig. 3a–c) as well as by staining against the proliferation marker Ki67 (Fig. 3d). No evidence for a survival/apoptosis related role of IVIG could be generated by means of staining for caspase-3 (data not shown). We could then show that IVIG treatment also affects Schwann cell morphology. As we previously demonstrated that, upon long-term suppression of the p57kip2 gene, Schwann cells can undergo cellular differentiation in absence of axons [20], we used this model for further examinations regarding a potential influence of IVIG on maturation processes. Along with an induction of myelin expression, differentiating primary rat Schwann cells also exhibit somatic and process growth [20]. We found that a significantly increased mean length of cellular processes could be observed in IVIG treated differentiating Schwann cells (Fig. 3e–g), after 5 days following p57kip2 suppression and 3 days of IVIG stimulation, while non-differentiating cells did not respond.

### IVIG stimulation induces myelin expression and activates maturation related signaling pathways

**Fig. 4:**
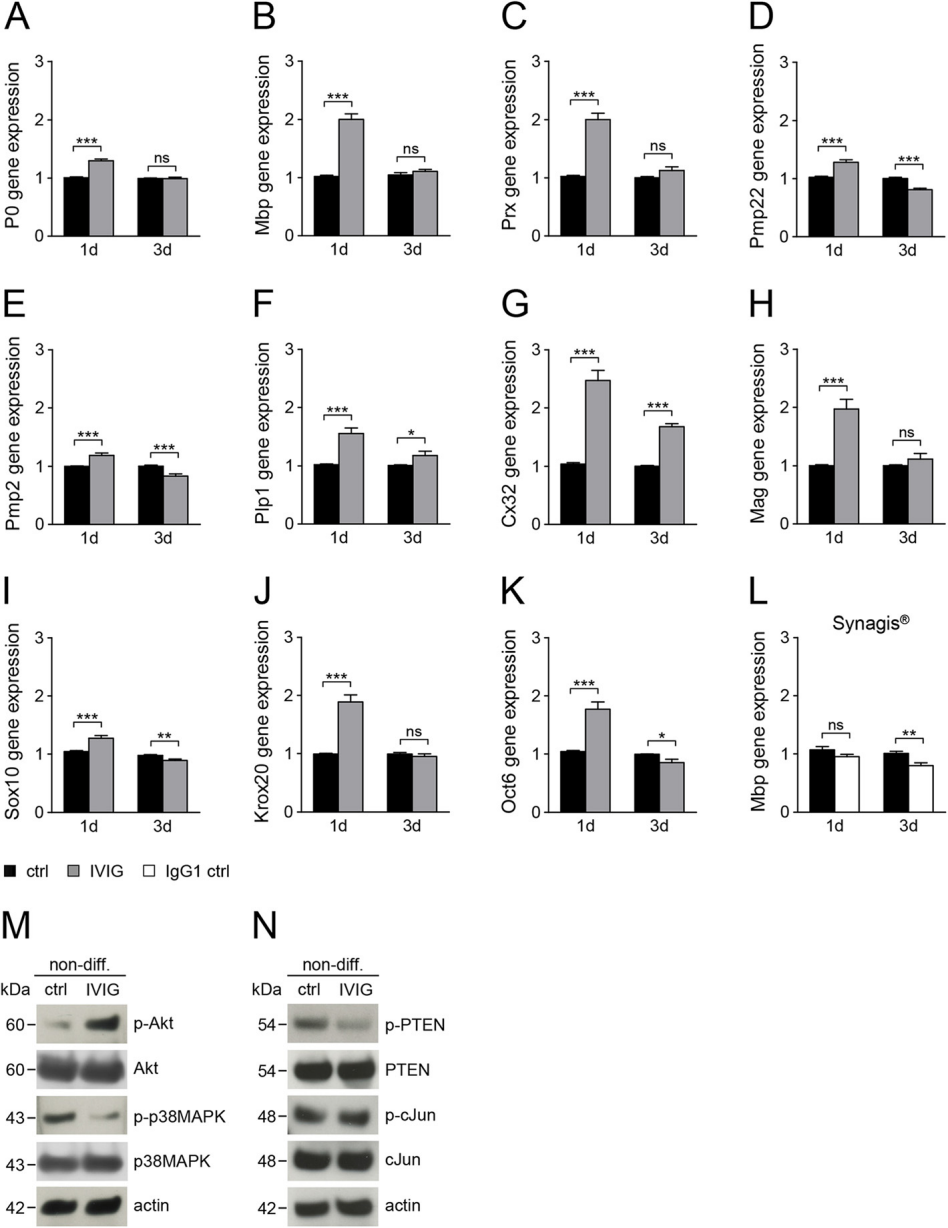
Short term IVIG effect on myelin gene expression and signaling molecules. **a**–**l** Gene expression measurements of non-differentiating Schwann cells (*non-diff*.) were performed after 1 and 3 days of IVIG, IgG1 control (*IgG1 ctrl*), and control buffer (*ctrl*) treatment. **a**–**h** Quantitative RT-PCR measurements of myelin gene expression showed consistent and significant upregulation of myelin genes after 1 day (*1d*) stimulation with 20 mg/ml dialysed IVIG (*gray bars*) as opposed to control buffer (*black bars*) stimulations. Myelin gene expression after 3 days (*3d*) IVIG stimulation was diminished. **i**–**k** Gene expression of important Schwann cell differentiation-related transcription factors was also increased upon 1 day IVIG stimulation. **l** Application of the human IgG1 Synagis (20 mg/ml, dialysed) did not affect MBP gene expression. GAPDH was used for normalization. **m**,**n** Western blot analysis (representative experiments) of signaling pathways related proteins revealed an increase in differentiation associated phospho-Akt (*p-Akt*) 1 day after IVIG treatment. Differentiation inhibitors such as phosphorylated forms of p38MAPK (**m**) or PTEN (**n**) were downregulated upon IVIG stimulation. Expression of cJun (**n**) was not changed after IVIG incubation. Actin was used for normalization and protein molecular weights are indicated in *kDa* (kilodalton). *t* test (*ns*, not significant, *p < 0.05, **p < 0.01, ***p < 0.001). *Error bars* represent SEM. Number of experiments: *n* = 18 for **a**, **b**, *n* = 7 for **c**, **d**, **g**, *n* = 4 for **e**, **h**, *n* = 11 for **f**, *n* = 5 for **i**, **j**, **k**, *n* = 3 for **l**, *n* = 2 for **m**, **n**

Next, we investigated whether IVIG treatment affects the expression of essential Schwann cell transcription factors, myelin (−related) genes, and signaling molecules. To this end, the transcriptional regulation of Mpz/P0, Mbp, Prx, Pmp22, Pmp2, Plp1, Cx32, and Mag (Fig. 4a–h) was examined. We found that stimulation of non-differentiating cultured Schwann cells with dialysed IVIG led to a significant upregulation of myelin gene expression within the first day of treatment, and that for most genes this induction was transient and restricted to the first 24 h of treatment (Fig. 4a–h). Importantly, when these cells were stimulated with three different human monoclonal IgG1 antibodies such as Synagis (Fig. 4l), Avastin, or Herceptin (data not shown), MBP (Fig. 4l), and Mpz/P0 (data not shown) expression was not increased, suggesting that the IVIG specific effect cannot be attributed to the presence of IgG1 with unrelated specificity. Consistent with the observed myelin induction, IVIG treatment also transiently boosted expression of transcription factors Sox10, Oct6, and Krox20/Egr2 (Fig. 4i–k), all of which were previously shown to be involved in the regulation of the myelination process [26].

**Fig. 5:**
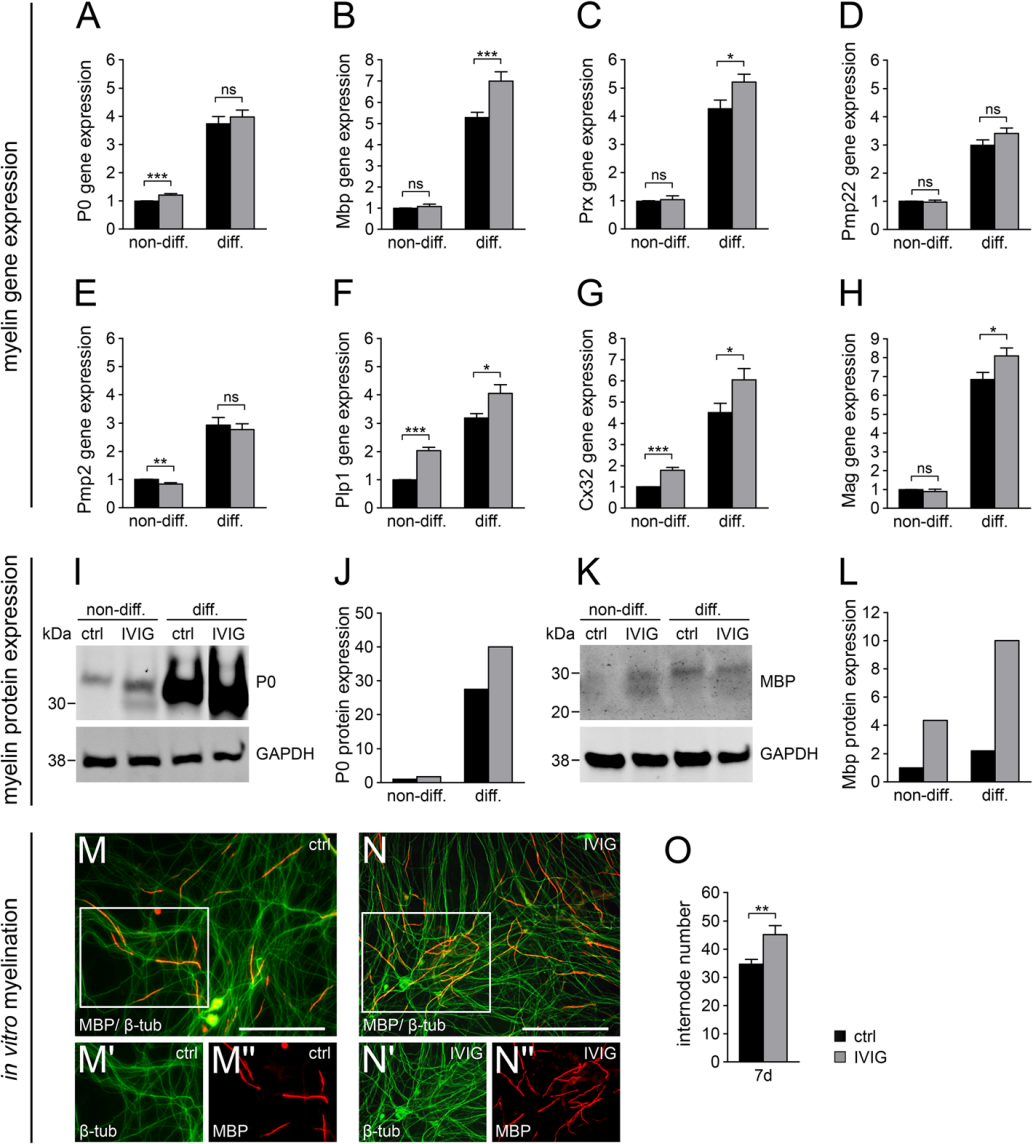
IVIG treatment induces myelin gene and protein expression. Differentiation competent Schwann cells respond to IVIG interaction by increasing their myelin gene (**a**–**h**) and protein expression (**i**–**l**). Two days after differentiation was initiated, 20mg/ml dialysed IVIG and control buffer (*ctrl*) were added to the culture medium. Treatment of differentiating (*diff*.) and non-differentiating (*non-diff*.) cells was pursued for 7 days, with one medium change at day 4. **a**–**h** Quantitative RT-PCR revealed significant upregulation of most myelin genes after 7 days stimulation with IVIG (*gray bars*) or control buffer (*black bars*). **i**–**j** Western blot analysis confirmed increased P0 (**i**, **j**) and MBP (**k**, **l**) protein expression after 7-day stimulation of differentiating and non-differentiating Schwann cells. **i**, **k** representative Western-blots, **j**, **l** corresponding Odyssey quantifications. Protein molecular weights are indicated in *kDa* (kilodalton). **m**–**o** Quantification of in vitro myelination revealed a significant increase of MBP positive internodes (*red*) after 7 days IVIG treatment (**o**); β-tubulin staining (*β-tub*) is visualized in *green*. **m**, **n** Representative photographs of myelinating neuron/glia cocultures treated with either control buffer (**m**) or IVIG (**n**). GAPDH was used as normalization control in all experiments. *t* test (*ns*, not significant, *p < 0.05, **p < 0.01, ***p < 0.001). *Error bars* represent SEM. Scale bar, 100 μm. *n* = 10 for **a**, *n* = 8 for **b**, *n* = 4 for **c**, *n* = 7 for **d**, **e**, *n* = 5 for **f**, **h**, *n* = 3 for **g**, *n* = 5 for **i**–**j**, **k**–**l**, *n* = 9 for **m**–**o**

Schwann cell differentiation and peripheral myelination depend on activation/phosphorylation of the serine-threonine kinase Akt, a process which is triggered by phosphatidylinositol 3,4,5-trisphosphate (PIP_3_) as product of the phosphatidylinositol 3-kinase (PIP_3_K) [27]. On the other hand, PTEN catalyzes the opposite reaction, the dephosphorylation of PIP_3_ to phosphatidylinositol 4,5-bisphosphate (PIP_2_), and can act thereby as indirect inhibitor of Akt activation and myelination [28]. Western blot analysis revealed increased levels of phosphorylated Akt (Fig. 4m) and downregulation of the phosphorylated PTEN protein (Fig. 4n) after 1 day of stimulation indicating that IVIG treatment initiates differentiation events. This was accompanied by downregulation of the phosphorylated form of p38 mitogen-activated protein kinase (p38MAPK; Fig. 4m), a prominent inhibitor of Schwann cell differentiation [29]. On the other hand, expression of cJun and phospho-cJun, an important negative regulator of Schwann cells differentiation [30, 31] was not altered upon IVIG treatment (Fig. 4n). Following the observation that differentiation incompetent Schwann cells transiently benefit from IVIG treatment, we then investigated to what degree such treatment can influence the differentiation process as such. For this purpose, we examined the myelin expression of differentiation competent (p57kip2 suppressed) cells upon IVIG treatment (Fig. 5a–l). Using quantitative RT-PCR, we could show that despite robust increases already after p57kip2 suppression (as previously shown in [20]), myelin gene expression was further induced by IVIG (Fig. 5a–h). Transcript levels of Mbp, Prx, Plp1, Cx32, and Mag were strongly induced upon IVIG stimulation, whereas Mpz/P0 and Pmp22 transcript levels (Fig. 5a, d) were only mildly induced and Pmp2 levels were found to be slightly downregulated (Fig. 5e). Note that myelin gene induction could be observed after a period of 7 days of IVIG treatment and was therefore not limited to early phases, as shown for differentiation incompetent cells (Fig. 4). As an exception, we found that upregulation of Plp1 and Cx32 was not restricted to early periods and could consistently be observed also in non-differentiating cells after 3 and 7 days of stimulation (Figs. 4f, g and 5f, g). These gene inductions were reflected by elevated levels of Mpz/P0 and MBP proteins, two of the most abundant proteins of peripheral myelin (Fig. 5i–l). We then studied the effects of IVIG application on axon/Schwann cell interactions by means of in vitro myelination (Fig. 5m–o). IVIG stimulation was done concomitant to the initiation of the myelination process and was performed for 7 days. In order to evaluate to which extent immunoglobulin treatment can modulate the generation of myelin sheaths, neuron/glia cocultures were stained using anti-MBP and anti-ß-tubulin antibodies (Fig. 5m, n) and MBP positive internodes were counted (Fig. 5o). This analysis revealed that in IVIG treated myelinating cocultures significantly more internodes were generated as compared to buffer treated cultures, indicating that IVIG exert a positive effect on myelin expression as well as on axonal wrapping process.

### IVIG induced expression of interleukin-18

**Fig. 6:**
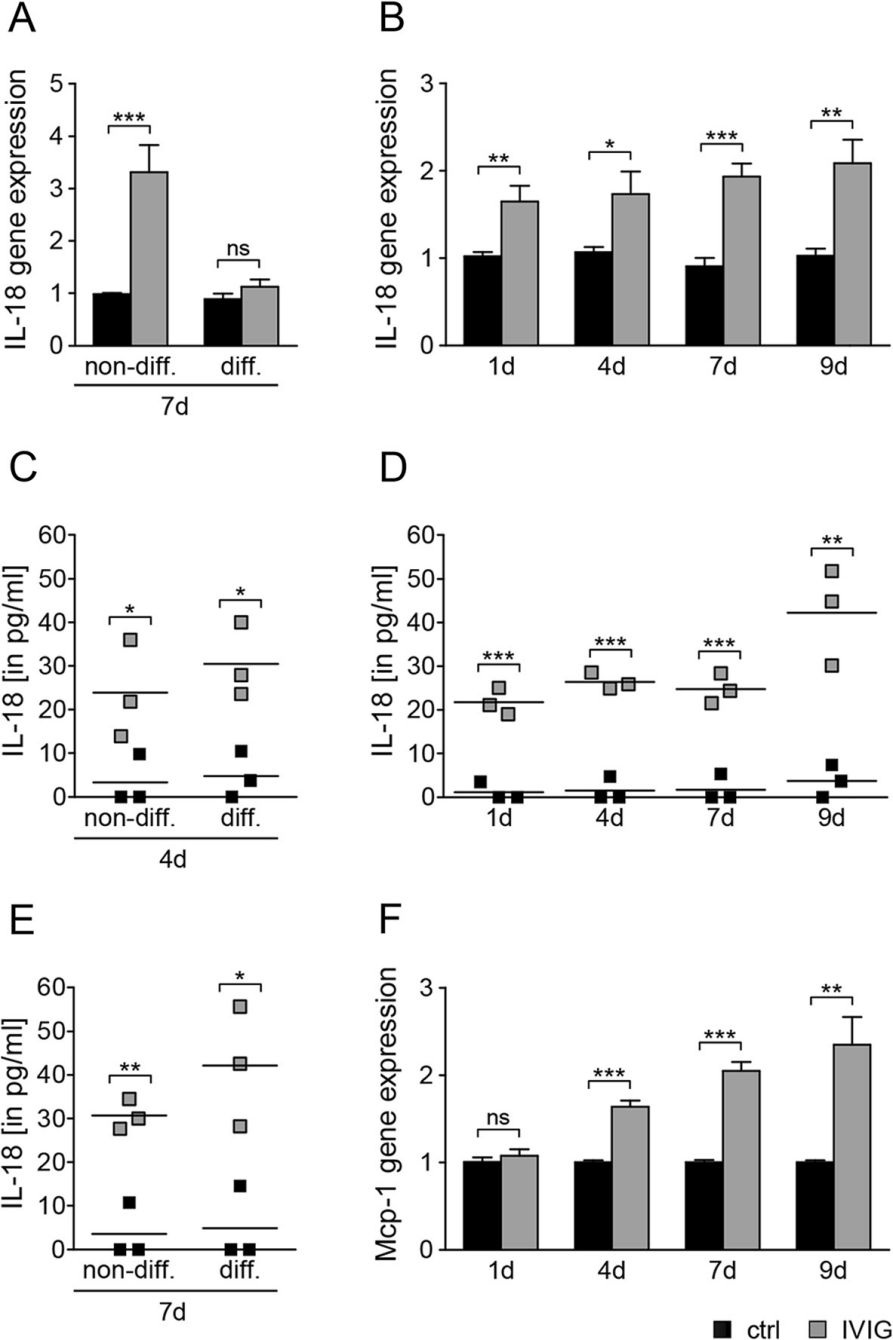
IVIG dependent expression and secretion of IL-18 and Mcp-1. **a**, **c**, **e** Two days after transfection, differentiating (*diff*.) and non-differentiating (*non-diff*.) Schwann cells were stimulated for up to 7 days (*7d*) with 20 mg/ml dialysed IVIG or control buffer (*ctrl*) with one medium exchange at day 4 (*4d*). At day 7, quantitative RT-PCR revealed a significant upregulation of IL-18 gene expression in non-differentiating cells (**a**) but not in differentiating cells. However, ELISA determination revealed elevated secreted IL-18 protein levels at both time points, *4d* and *7d*, after IVIG stimulation (**c**, **e**). **b**, **d** Similarly, induced IL-18 gene and protein expression levels could be observed when naive (non-transfected and therefore non-differentiating) Schwann cells were IVIG stimulated as shown on time points *1d*, *4d*, *7d*, and *9d*. Of note, *4d* medium was collected before the medium change. **f** Mcp-1 gene expression levels were also upregulated after *4d*, *7d*, and *9d* of IVIG stimulation. GAPDH was used as reference gene (**a**, **b**, **f**). *t* test (*ns*, not significant, *p < 0.05, **p < 0.01, ***p < 0.001). *Error bars* represent SEM. *n* = 7 for **a**, *n* = 3 for **b**, **f**, *n* = 3 for **c**, **d**, **e**

In order to identify further transcriptional changes induced by IVIG treatment in rat Schwann cells, a GeneChip Array analysis was performed on non-differentiating and differentiating Schwann cells which have been stimulated by IVIG or control buffer for 7 days. A detailed description of IVIG regulated genes will be described elsewhere; however, we found that one of the most prominently upregulated genes was interleukin-18 (IL-18), a proinflammatory cytokine belonging to the IL-1 family [32]. According to the GeneChip data, IL-18 was significantly upregulated in non-differentiating Schwann cells. We therefore investigated this finding in greater detail and validated transcript levels by means of quantitative real-time RT-PCR (Fig. 6a). This analysis confirmed strong induction in non-differentiating cells as well as that IVIG treatment can induce IL-18 expression over a period of at least 9 days (Fig. 6b). Moreover, by means of ELISA, we could show that cultured Schwann cells also secrete elevated IL-18 levels at days 1, 4, 7, and 9 upon IVIG stimulation (Fig. 6d). Of note, although at late time points differentiating Schwann cells were not showing an IVIG dependent increase in IL-18 transcript levels (Fig. 6a), IL-18 protein levels were elevated in both Schwann cell populations after 4 and 7 days (Fig. 6c, e). In addition, we examined the transcriptional regulation of monocyte attractant protein-1 (Mcp-1), a further proinflammatory molecule secreted by Schwann cells, which is involved in macrophage attraction/activation during Wallerian degeneration [33, 34] as well as in genetically induced demyelination [35]. We found Mcp-1 transcript levels to be consistently upregulated at later time points during IVIG stimulation of non-differentiating Schwann cells (Fig. 6f).

### Schwann cell promoted neurite outgrowth

**Fig. 7:**
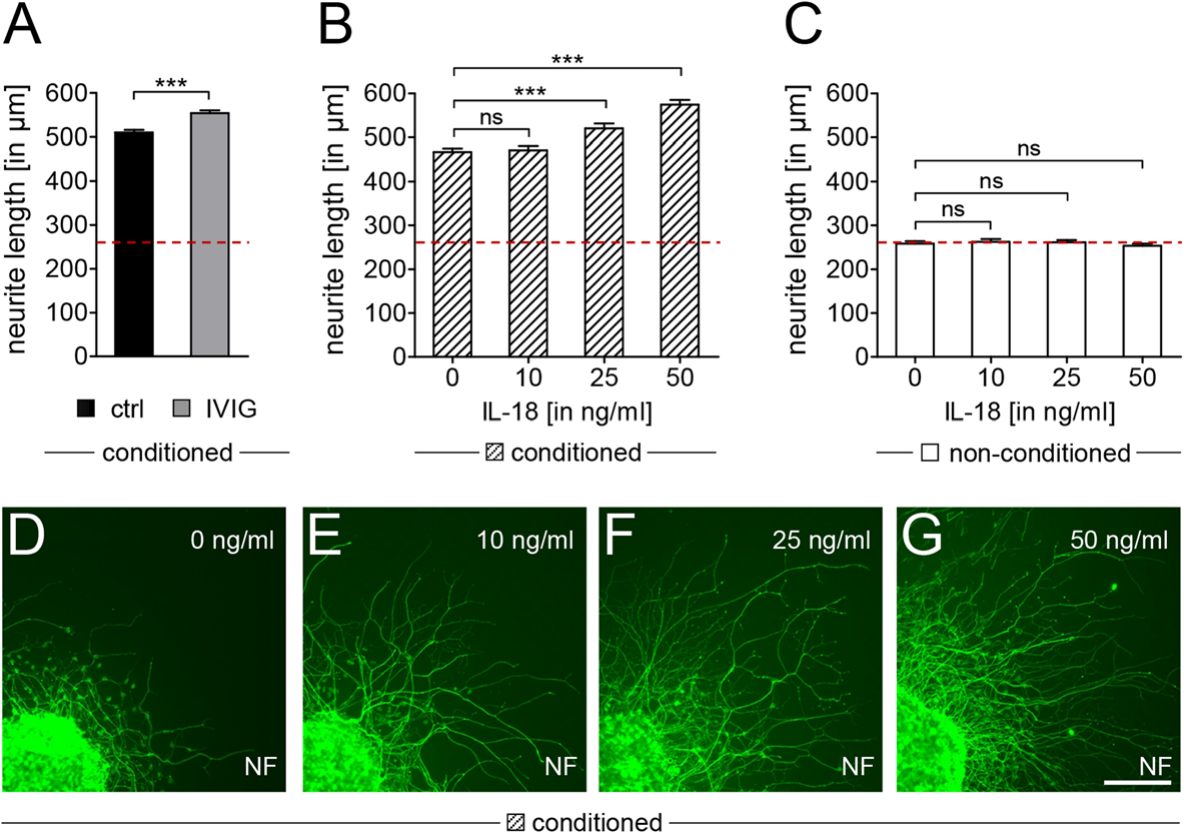
Schwann cell dependent promotion of axonal outgrowth. **a**–**g** Average neurite growth lengths were determined 24 h after incubation of rat DRG explants in culture medium of Schwann cells treated with control buffer (*ctrl*), IVIG, or different IL-18 concentrations. Three different culture media were used: **a** culture medium conditioned by control buffer treated Schwann cells (*black bar*) and by cells stimulated with 20 mg/ml dialysed IVIG (*gray bar*) for 3 days; **b** culture medium conditioned by Schwann cells treated with IL-18 over 3 days and **c** culture medium directly supplemented with IL-18 (*non-conditioned*). Quantification of neurite lengths (*NF*-positive) from the DRG core to the outer rim revealed extensive radial neurite outgrowth in Schwann cell conditioned media (**a**, **b**) in comparison to non-conditioned culture media (**c**). Treatment of Schwann cells with IVIG further increased the neuritic growth (**a**), and media conditioned by IL-18 treated Schwann cells also showed a positive, concentration dependent effect on outgrowth (**b**), whereas direct addition of IL-18 to the culture medium did not exert any effect (**c**). **d**–**g** Representative DRG explants cultured in medium conditioned by Schwann cell that were stimulated by different IL-18 concentrations; neurofilament (*NF*) staining is visualized in *green. t* test (*ns*, not significant, ***p < 0.001). *Error bars* represent SEM. Scale bar, 200 μm. *n* = 3 for **a**, *n* = 5 for **b**, **c**, per experiment and condition 6–12 explants were evaluated. *Red line* marks the average neurite length in response to non-conditioned medium (0 ng/ml IL-18 in **c**)

We observed that conditioned medium from Schwann cells treated with IVIG over 3 days could significantly induce neurite outgrowth of sensory neurons (Fig. 7a) as revealed by in vitro neurite growth assays based on dorsal root ganglion (DRG) explant cultures as previously shown [36]. Considering the here detected IL-18 expression by Schwann cells upon IVIG stimulation, we investigated whether this cytokine might contribute to this effect. To test this possibility, DRG explants were given medium that has been conditioned over 3 days by IL-18 stimulated Schwann cells (Fig. 7b), or supplied with medium freshly supplemented with recombinant IL-18 (Fig. 7c). Axonal outgrowth was measured 24 h after plating by determining the average length of neurofilament-positive neurites. As expected, Schwann cell conditioned medium improved neurite outgrowth (compare levels in Fig. 7a, b, vs. c; red dotted line). Importantly, this positive growth effect was further potentiated in a dose dependent way when medium was conditioned by IL-18 treated Schwann cells (Fig. 7b, d–g). In cultures treated with non-conditioned Schwann cell medium and freshly added IL-18, neurite growth was not affected (Fig. 7c).

## Discussion

IVIG have been used for more than two decades for the treatment of demyelinating immune system disorders, especially inflammatory neuropathies, and the possibly underlying immunomodulatory effects have extensively been described [9]. However, a direct effect of pooled immunoglobulins on glial cells of the peripheral nervous system, hence a potential regeneration associated role, has not been shown so far. In this study, we could demonstrate that IVIG specifically bind to the surface of primary Schwann cells and we also detected expression of a high affinity Fc receptor on these cells. As a result of IVIG binding, Schwann cell maturation was initiated and promoted, and in the presence of axons an increased number of internodes could be detected. Note that in non-differentiating Schwann cells, IVIG were nevertheless able to induce robust transient molecular reactions but could not maintain long-term alterations, indicating that the need to either remove intrinsic inhibitory components [37] or to provide axonal contact cannot be completely overcome by this treatment.

In addition, we here demonstrate for the first time that Schwann cells secrete IL-18 and that production of this cytokine is boosted upon IVIG stimulation. Functional studies using DRG explants then revealed that IVIG and IL-18 stimulated Schwann cells can both support axonal outgrowth whereas direct IL-18 treatment of neurons showed no effect. This suggests a fundamental contribution of this cytokine to restoration processes of injured and diseased nerves. Whether IL-18 is solely responsible for the IVIG-mediated neuritic growth effect remains to be shown. It can therefore be concluded at this point that IVIG cannot only positively influence Schwann cell differentiation processes but that their regenerative potential also benefits from this stimulation. As an IL-1 family member, IL-18 is considered a proinflammatory cytokine, which is not only expressed by immune competent cells such as T lymphocytes and macrophages but also CNS cells such as microglia and astrocytes [38]. In the PNS, elevated levels of IL-18 were shown to co-localize with activated macrophages in inflamed nerve roots of EAN rats, as well as in serum and cerebrospinal fluid of GBS patients [39]. IL-18 was also found to be transiently induced after peripheral nerve injury. In these nerves, IL-18 expression was assigned to macrophages and supposed to mediate macrophage engagement for effective myelin uptake [40]. In light of our findings, IL-18 might therefore exert additional neuroprotective and neurorestorative roles also in inflammatory nerve conditions. It will be of interest to learn in future studies to what degree IL-18 can change the glial secretome and to identify the nature of released axonal growth factors.

On the other hand, an IVIG enforced/instructed interaction between Schwann cells and macrophages is further suggested by the observed induction of the Mcp-1. This chemokine is known to attract macrophages to the site of injury in Wallerian degeneration and in CMT models thus displaying a beneficial effect on the inflammation process after nerve injury [41].

The underlying mode of action, especially concerning the exact way immunoglobulins interact with Schwann cell surface proteins, needs to be further explored and might include multiple different mechanisms. Currently, we hypothesize that both ways of surface recognition are possible. On the one hand, immunoglobulins could recognize specific Schwann cell epitopes, whereas on the other hand, Fc receptor binding could also occur, similar to the described IVIG interactions with components of the immune system [9]. Future studies must include a determination to what degree glial reactions depend on IgG monomers, dimers, or oligomers as they can occur in solution as well as surface bound. According to our SEC data, IgG dimers are present but their fraction did not substantially change upon dialysis over time. Detection of CD64 receptor for high affinity IgG binding on Schwann cells, as well the finding that IVIG-derived F(ab′)_2_ fragments can also specifically interact with the cell surface, support such a dual mode of action. Although identification of maturation- and regeneration-associated glial epitopes would constitute a major step towards refined nerve repair approaches, such investigations must be reserved for future studies and will most likely imply state of the art biochemical analyses. It is therefore conceivable that a constellation similar to oligodendroglial cells could develop, where expression of an Fc receptor for IgM on mouse oligodendrocytes was initially reported [18]. Whereas further studies showed that pentameric IgM bind to oligodendroglial cells and promote their differentiation, the recombinant human autoantibody rHIgM22, of which vitronectin/fibronectin receptor αvβ3 was identified as a potential target, emerged from these studies and has now entered clinical trials [42, 43]. Importantly, although polyclonal IgM molecules turned out to be more potent inducers of oligodendroglial differentiation and remyelination, human IVIG also promoted this process [15–17]. Nevertheless, one should be aware of possible xenoreactive effects since in the current study we used human immunoglobulin preparations and examined their effect on rat and mouse cell culture models. Future translations of our findings will therefore include the study of IVIG’s role in vivo as well as investigations using either rodent immunoglobulins or human Schwann cells.

It is of interest to see that early studies already described Fcgr3/CD16 receptor expression by human Schwann cells [44, 23, 24]. Nevertheless, CD16 expression was found to be dependent on axonal contact and at lower levels when compared to macrophages [45, 25]. This is in line with our data showing that differentiating Schwann cells exhibited significantly higher CD64 receptor levels as compared to non-differentiating cells. Whether or not IVIG directly interact with CD64 on the Schwann cell surface and thus this interaction contributes to the glial immunocompetence [4] remains to be determined in future examinations. In order to determine the exact mode of IVIG action, digested/modified fractions of IVIG could be used allowing only one possible way of surface binding. This along with investigations directed to determine immunoglobulin complex formation on cell surfaces (vs. in solution) could be of help to exclude the possibility that IgG bind to non-biogenic surfaces and thus mimic immune complexes.

Independent from the description of active components and their appropriate partner proteins on Schwann cells, the observation that immunoglobulin/glial cell interactions take place and that, as a consequence, cellular maturation is induced and a regenerative phenotype is promoted, is of interest as such. Our observations might also reflect recent findings on Wallerian degeneration related intrinsic (auto-) antibodies, contributing to successful nerve repair [12]. However, a different mode of action was suggested as the authors found immunoglobulins to bind degenerating myelin and to promote macrophage-dependent clearance. Similar immune-related mechanisms have been proposed in CMT1 models of inherited neuropathies [41].

## Conclusions

Our findings might pave the way for future nerve repair therapies, favored by a high safety profile of IVIG. It therefore remains to be shown in future studies to what degree endogenous as well as therapeutically applied immunoglobulins exert combinatorial effects including local immunomodulation, effective myelin opsonisation and true glial modulation for functional nerve restoration. Given the central role of Schwann cells for regeneration in most if not all nerve pathologies, this strongly suggest that IVIG could be used as supportive treatment for endogenous nerve repair processes. As this is still an unmet therapeutic goal, a number of patients with various nerve pathologies could benefit from such a treatment.

## Abbreviations

PNS: peripheral nervous system
IVIG: intravenous immunoglobulins
IgG: immunogloblin G
Fc: fragment crystallisable
IgM: immunogloblin M
EAN: experimental autoimmune neuritis
CNS: central nervous system
PDL: poly-D-lysine
FBS: fetal bovine serum
BrdU: 5-bromo-2′-deoxy-uridine
DRG: dorsal root ganglion
E: embryonic day
PFA: paraformaldehyde
MBP: myelin basic protein
β-tub: β-tubulin
NF: neurofilament
rrIL-18: rat recombinant interleukin-18
Fab: fragment antigen-binding
MWCO: molecular weight cut-off
DAPI: 4′,6-diamidino-2-phenylindole
GAPDH: glyceraldehyde-3-phosphate dehydrogenase
ODC: ornithine decarboxylase
Pmp22: peripheral myelin protein-22
Sox10: SRY-related HMGbox-10
Krox20/Egr2: early growth response-2
Oct6: octamer-binding transcription factor-6
Mpz/P0: myelin protein zero
Prx: periaxin
Pmp2: peripheral myelin protein 2
Plp1: proteolipid protein 1
Cx32: connexion 32
Mag: myelin associated glycoprotein
Mcp-1: monocyte attractant protein
IL-18: interleukin-18
Fcgr1a/CD64: Fc fragment of IgG, high affinity 1a, receptor
NRS: normal rabbit serum
NGS: normal goat serum
BSA: bovine serum albumin
PBS: phosphate-buffered saline
HRP: horse-radish-peroxidase
Akt: serine-threonine kinase
PTEN: phosphatase and tensin homolog
p38MAPK: p38 mitogen-activated protein kinase
non-diff.: non-differentiating
diff.: differentiating
ctrl: control buffer
SEM: standard error of the mean
*n*: number of experiments
